# Molecular modeling of complete tertiary structure of pyrin and influence of mutations on it

**DOI:** 10.1186/1546-0096-13-S1-O14

**Published:** 2015-09-28

**Authors:** G Arakelov, K Nazaryan

**Affiliations:** 1 Russian-Armenian (Slavonic) University, Bioengineering and Bioinformatics, Yerevan, Armenia; 2 Institute of Molecular Biology of the National Academy of Sciences of the Republic of Armenia, Laboratory of Computational Modeling of Biological Processes, Yerevan, Armenia

## Introduction

Pyrin protein is the product of the MEFV gene, mutations in which cause the manifestation of Familial Mediterranean Fever (FMF). Complete tertiary structure of pyrin and the effects of mutations on it are still experimentally not studied. Mutations - E148Q, M680I, M694V, M694I, V726A, A744S and R761H of pyrin induce manifestation of the most widespread and severe forms of FMF. One striking feature of FMF is the phenomenon of complex allele mutations. In case of complex allele mutations, the pyrin protein will contain more than one mutated amino acids. From currently known complex allele mutations there are those that combine the most widespread and severe single mutations - M680I-M694I, E148Q-M694V, E148Q-M694I, E148Q-V726A, E148Q-A744S, E148Q-R761H, E148Q-V726A-R761H. In complex alleles, one mutation may have a modifying effect on the other one. The other striking feature of FMF is the fact that despite the fact that the FMF is considered as an autosomal recessive autoinflammatory syndrome, were detected mutations - T577N и T577S for which was proved autosomal dominant inheritance. Understanding the correlation between the FMF phenotype and genotype is further obscured by the existence of complex allele and dominant mutations. Therefore, we suggest that computational modeling of native and mutated pyrin complete tertiary structure and their comparative investigation will help to understand the effects of abovementioned mutations on pyrin and on FMF manifestation in general.

## Objectives

From abovementioned the goal of current study was to detect the effect of mutations on the complete tertiary structure of the pyrin protein for clarifying its functions in the autoinflammatory processes and in the pathogenesis of FMF.

To achieve our objectives the following tasks were set:

1) Develop a computer model of the complete tertiary structure of pyrn and validate the obtained model.

2) Develop a computer model of the tertiary structure of pyrin with single, dominant and complex allele mutations.

3) Analyze the impact of mutations on the tertiary structure of pyrin.

4) Compare the effects of dominant and complex allele mutations influence with the single recessive mutations effects in order to clarify the effect of dominant and complex allele mutations to the FMF.

## Methods

Molecular modeling of pyrin native tertiary structure and its mutant variations was carried out using the software package ROSETTA 3.5 using de novo and threading modeling methods. For validation and to determine the correctness of the obtained model VADAR and RESPROX programs was used. Models visualization and analysis were performed using VMD program. These software packages have been used in the operating system Linux, by 24-nood computer cluster and HPC of M.V. Lomonosov Moscow State University.

## Results

Using de novo and threading modeling methods was obtained 1000000 models of the pyrin protein tertiary structure. Then was carried out validation of the pyrin structure best model and assessment of its stereochemical correctness. As a result, it was found that obtained model have a resolution of 1,6 Å, which is a high resolution of stereochemistry correctness for a such large protein. In order to study the effect of mutations on the tertiary structure of pyrin were modeled tertiary structures for the abovementioned single, dominant and complex allele mutations, using homology modeling method. For each mutation 10000 models have been obtained. After which the analysis and comparison of the native and mutated tertiary structures of pyrin were carried out, which showed that mutations lead to structural rearrangements such as: transition loop - β-sheet, loop - α-helix, β-sheet - loop, β-sheeted - α-helix, α-helix - loop and α-helix - β-sheet. Also were observed rearrangements leading to elongation of β-sheets and α-helices, to a shortening of β-sheets and α-helices and to partition of one large β-sheet into two smaller ones.

## Conclusion

From obtained results the following conclusions can be made:

**1)** Have been obtained the model of pyrin complete tertiary structure with a resolution of 1.6Å.

**2)** It was found that all the studied mutations lead to structural rearrangements affecting on the tertiary structure of pyrin.

**3)** in spite of the domain affiliation of studied mutations, they lead to structural rearrangements in other parts of the pyrin also.

